# A New Strategy to Generate Functional Insulin-Producing Cell Lines by Somatic Gene Transfer into Pancreatic Progenitors

**DOI:** 10.1371/journal.pone.0004731

**Published:** 2009-03-06

**Authors:** Philippe Ravassard, Emilie Bricout-Neveu, Yasmine Hazhouz, Severine Pechberty, Jacques Mallet, Paul Czernichow, Raphael Scharfmann

**Affiliations:** 1 Biotechnology & Biotherapy group Centre de Recherche Institut du Cerveau et de la Moelle CNRS UMR7225, INSERM UMRS795, Université Pierre et Marie Curie, Paris, France; 2 Endocells, Paris, France; 3 INSERM U845, Centre de Recherche Croissance et Signalisation, Université Paris Descartes, Faculté de Médecine, Hôpital Necker, Paris, France; University of Bremen, Germany

## Abstract

**Background:**

There is increasing interest in developing human cell lines to be used to better understand cell biology, but also for drug screening, toxicology analysis and future cell therapy. In the endocrine pancreatic field, functional human beta cell lines are extremely scarce. On the other hand, rodent insulin producing beta cells have been generated during the past years with great success. Many of such cell lines were produced by using transgenic mice expressing SV40T antigen under the control of the insulin promoter, an approach clearly inadequate in human. Our objective was to develop and validate in rodent an alternative transgenic-like approach, applicable to human tissue, by performing somatic gene transfer into pancreatic progenitors that will develop into beta cells.

**Methods and Findings:**

In this study, rat embryonic pancreases were transduced with recombinant lentiviral vector expressing the SV40T antigen under the control of the insulin promoter. Transduced tissues were next transplanted under the kidney capsule of immuno-incompetent mice allowing insulinoma development from which beta cell lines were established. Gene expression profile, insulin content and glucose dependent secretion, normalization of glycemia upon transplantation into diabetic mice validated the approach to generate beta cell lines.

**Conclusions:**

Somatic gene transfer into pancreatic progenitors represents an alternative strategy to generate functional beta cell lines in rodent. Moreover, this approach can be generalized to derive cells lines from various tissues and most importantly from tissues of human origin.

## Introduction

Islets of Langerhans are micro-organs scattered throughout the pancreas, and responsible for synthesizing and secreting pancreatic hormones. They produce hormones such as insulin (beta cells), glucagon (alpha cells) somatostatin (delta cells) and pancreatic polypeptide (PP cells). Insulin-secreting beta cells play a major role and are involved in the development of diabetes. Type 1 diabetes results from autoimmune destruction of pancreatic beta cells, while type 2 diabetes is caused by a combination of insulin resistance and inadequate insulin secretion. Thus, in both type 1 and type 2 diabetes, the functional pancreatic beta cell mass is not sufficient to control glycemia. Over the past decades research in the beta cell field profited from the establishment of insulin-secreting cell lines. Many rodent cell lines were produced from adult beta cells such as RIN and INS1 cells derived from x-ray induced adult rat insulinoma [1], [2] and HIT cells generated by simian virus 40 transformation of adult hamster islet cells [3]. Others were established from transgenic mice expressing SV40T antigen under the control of the insulin promoter [4]–[9]. Such rodent cell lines were extremely useful for a better understanding of beta cell biology. Attempts were next made to generate human beta cell lines using the strategies developed for rodent cell lines. Unfortunately, the majority of the attempts were not successful.

In the present work, our objective was to validate alternative transgenic approaches to generate beta cell lines using a procedure that can be transferred to human. Specifically, we asked whether beta cell lines can be generated from embryonic pancreatic explants by somatic gene transfer into pancreatic progenitor cells that will differentiate into beta cells.

For this purpose, we transduced immature rat pancreatic tissues with recombinant lentiviral vector expressing SV40T antigen under the control of the insulin promoter. Our data demonstrate that such vector can transduce pancreatic stem/progenitors, that will differentiate into beta cells expressing the SV40T antigen and form insulinoma from which beta cell lines can be derived. This strategy that mimics transgenic-like approach represents a new process to generate beta cell lines that can be applicable to human tissue.

## Results

### Tumour formation from rat embryonic pancreases transduced with recombinant lentiviruses expressing the SV40T antigen under the control of the insulin promoter

**Figure 1:**
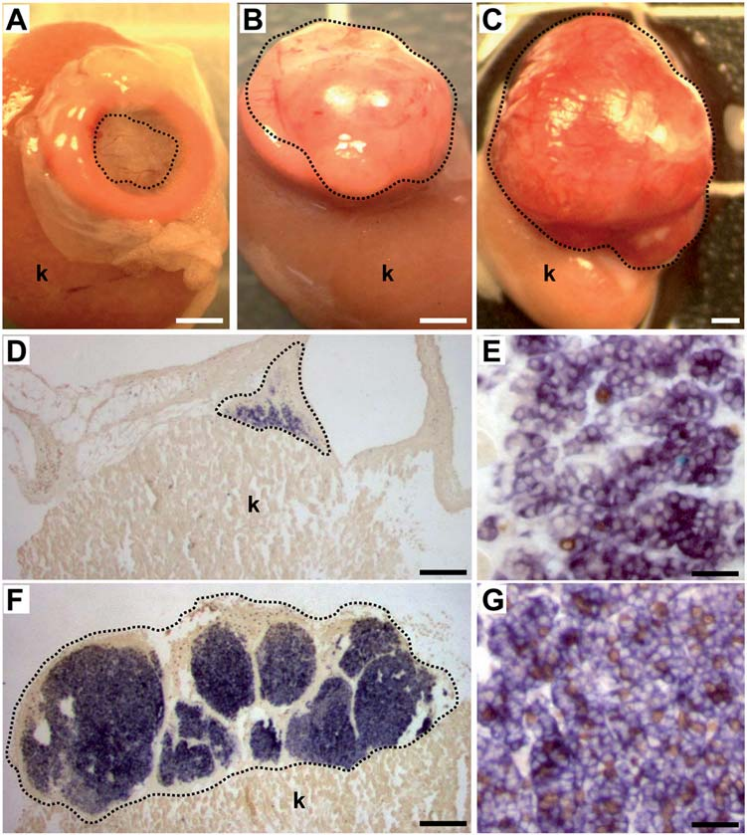
Development of the infected tissue after transplantation. A–C: Pancreatic epithelia were transduced with pTrip ΔU3.RIP405-eGFP (A) or pTRIP ΔU3.RIP405-SV40T (B, C), transplanted and analyzed one (A, B) or three months later (C). D–G: Insulin detection by in situ hybridization (blue) on 10 µm sections on grafts removed one month after transplantation. In D and E, grafts was transduced with pTrip ΔU3.RIP405-eGFP. In F and G, graft was transduced with pTRIP ΔU3.RIP405-SV40T. E, G: Double staining for insulin (blue) and BrdU (brown). Pancreatic tissue is circled with a dotted line, k: kidney. Scale bars: A–C 2 mm; D, F 1 mm; E, G 25 µm.

**Figure 2:**
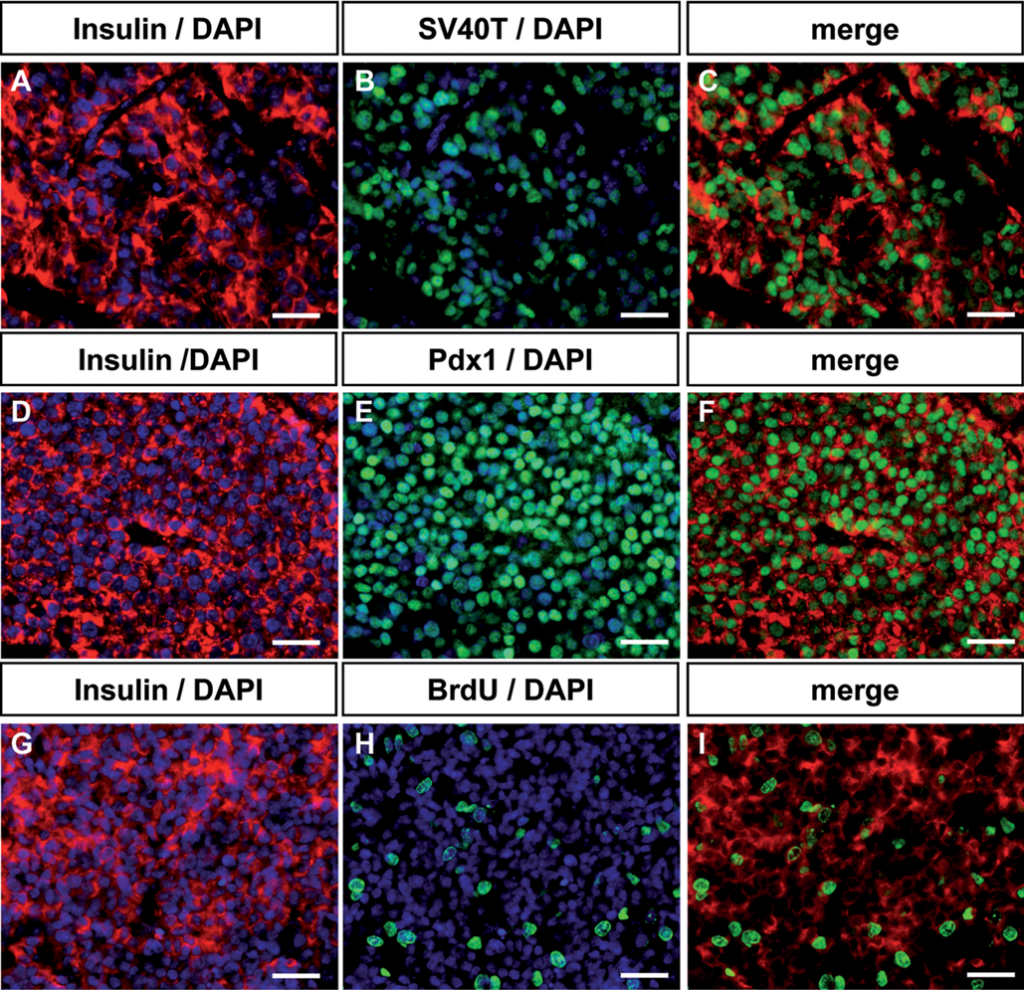
Immunohistochemical analysis of grafts developed in Scid mice. A–C: Staining for insulin (red), SV40T (green) and DAPI (blue); D–F: Staining for insulin (red), Pdx1 (green) and DAPI (blue); G–I: Staining for insulin (red), BrdU (green) and DAPI (blue). Scale bars: 25 µm

We previously demonstrated that mature insulin producing cells can be stably modified by transduction of pancreatic progenitors with recombinant lentiviruses expressing eGFP under the control of the insulin promoter [10]. Here, we asked whether such an approach could be used to generate beta cell lines by transduction of pancreatic progenitors with recombinant lentiviruses expressing the SV40T antigen under the control of the insulin promoter. Lentiviral vectors expressing either SV40T antigen (pTRIP ΔU3.RIP405-SV40T) or eGFP (pTrip ΔU3.RIP405-eGFP) under the control of a 405 bp fragment of the rat insulin promoter were used to transduce immature E13 rat embryonic pancreases that were transplanted under the kidney capsule of immuno-incompetent *scid* mice. One month later, the grafted tissues were removed and analyzed. The size of the grafted tissue was enlarged when tissues were transduced with viral vectors expressing SV40T antigen when compared to transduction with viral vectors expressing eGFP (Fig. 1, compare panels A and B). When SV40T antigen transduced pancreases were removed 3 months after transplantation, the size of the tissue was even higher (Fig. 1C). In the grafts, insulin expression was monitored by in situ hybridization. While some *insulin*-expressing cells were detected in pancreases transduced with viral vectors expressing eGFP, their number was hugely increased in pancreases transduced with SV40T expressing viral vectors (Fig. 1, compare panels D and F). Similarly, proliferation of *insulin*-expressing cells detected by BrdU incorporation was largely increased in grafts transduced with SV40T compared to eGFP expressing viral vectors (Fig. 1, compare panels E and G). Insulin expression derived from pancreases transduced with viral vectors expressing SV40T antigen was next analyzed at the protein level. As expected, insulin-positive cells expressed SV40T antigen (Fig. 2, panels A–C). They also expressed Pdx1, a transcription factor crucial for beta cell development and function [11] (Fig. 2, panels D–F) and incorporate BrdU (Fig. 2, panels G–I).

### Establishment of rat pancreatic beta cell lines

**Figure 3:**
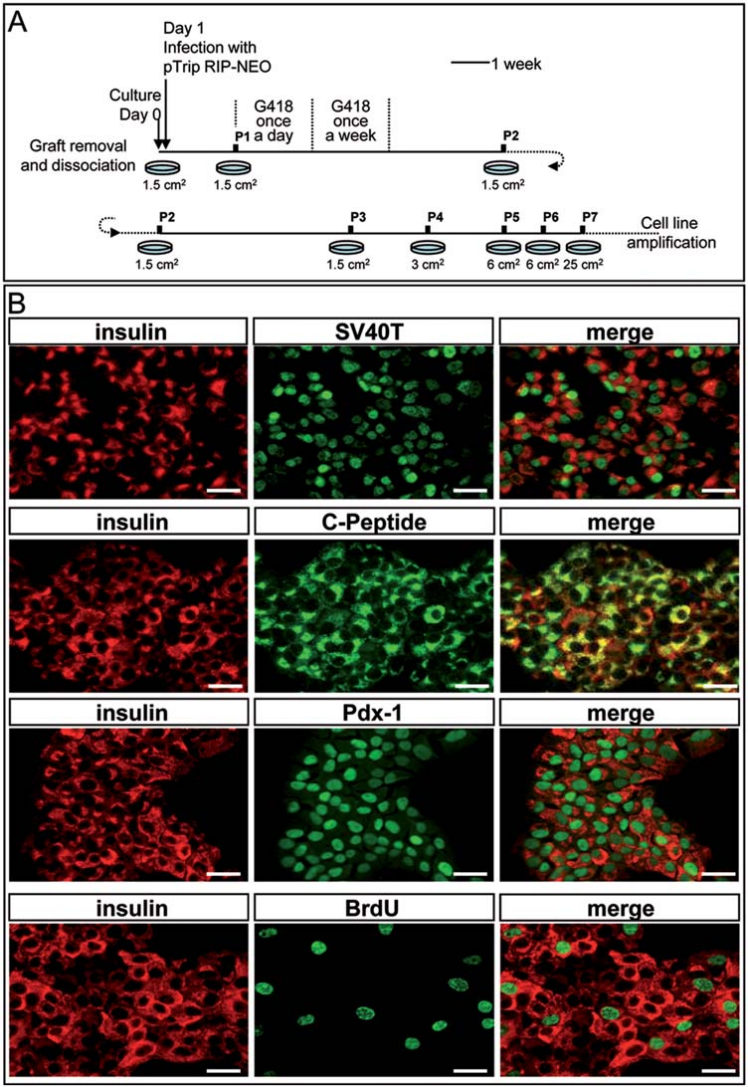
Immunocytochemical Characterization of RYAS41 cells. A: Schematic representation of the culture procedure used to derive the RYAS41 cell line. P represents passage number. Surface of the culture well is indicated below the time line. B: Coexpression of insulin (red) and SV40T (green); insulin (red) and c-peptide (green), insulin (red) and Pdx1 (green); and double staining for insulin (red) and BrdU (green). Scale bars: 25 µm.

To establish pancreatic beta cell lines, grafts were removed, dissociated and transduced with viral vectors expressing the neomycin resistance gene under the control of the insulin promoter for further selection of insulin-transcribing cells by culture in the presence of G418 (Fig. 3A). Using this protocol, different cell lines were established and one of them, RYAS41 was further analyzed. As shown in Fig. 3B, RYAS41 cells expressed insulin, C-peptide and SV40T antigen. They also express the nuclear transcription factor Pdx1 and proliferate based on their capacity to incorporate BrdU. Such cell lines can be frozen and thawed and its features are stable at least until passage 54.

### RYAS41 cell line expresses beta cell specific markers and secretes insulin in response to glucose stimulation

**Figure 4:**
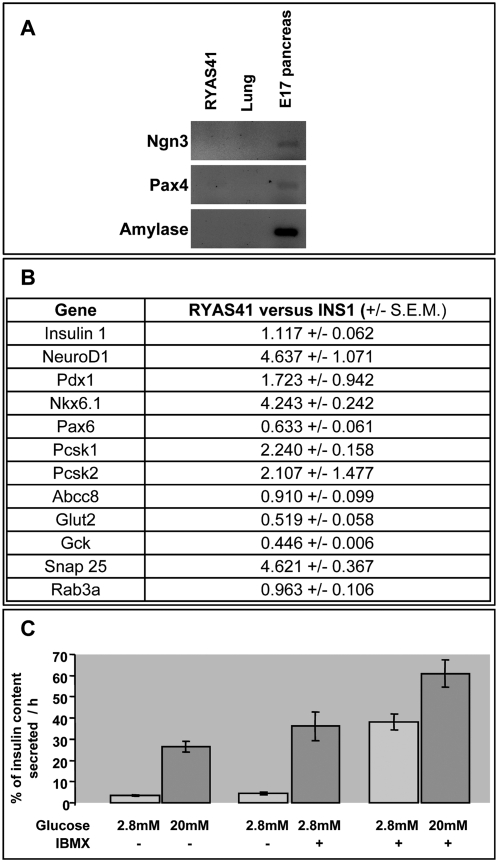
Gene expression profile in RYAS41 and glucose-stimulated insulin secretion. A: Semi-quantitative RT PCR comparison between RYAS41, lung (negative control) and pancreas (positive control) from E17 rat embryos. PCR products after 40 amplification cycles are analyzed on a 2% agarose gel. B: CT (threshold cycle) value are normalized to cyclophilin and presented as fold increase compared to 832/13 INS-1 cells. Values are means+/−S.E.M. of Q-PCR performed in duplicates from 3 independent RNA extractions. C: RYAS41 secrete insulin in response to glucose stimulation. Insulin secreted into the medium is presented as % of insulin content secreted per hour. Values are means+/−S.E.M. of three independent cell cultures.

RYAS41 lack expression of *Ngn3* and *Pax4* (Fig. 4A), two transcription factors expressed in pancreatic endocrine progenitor cells [12], [13] and either absent [13] or expressed at very low levels [14] in mature beta cells. In addition, expression of acinar marker such as amylase was not detected in RYAS41 cells (Fig. 4A).

We next compared by quantitative RT PCR the expression profile of RYAS41 to the well characterized 832/13 INS-1 cells [15]. We analyzed the expression of three transcription factors *Pdx1*, *NeuroD1* and *Nkx6.1* and many genes important for beta cell function such as Pcsk1, Pcsk2, Abcc8, Glut2, Glucokinase, Snap25 and Rab3A. For all markers tested, the expression in RYAS41 cells was in the same range as the one found in 832/13 INS-1 cells (Fig. 4B). Moreover, insulin1 mRNA expression was identical in RYAS41 cells when compared to 832/13 INS-1 cells (Fig. 4B). At the protein level, the insulin content per cell was 2.1 fold lower in RYAS41 cells when compared to 832/13 INS-1 cells (0.32+/−0.04 vs 0.69+/−0.07 pg of insulin per cell respectively). When normalized to total protein, RYAS41 and 832/13 INS-1 cells contain 2.87+/−0.6 and 6.92+/−0.81 ng insulin / µg protein respectively.

Finally, insulin secretion of RYAS41 cells was analyzed after glucose stimulation in the presence or absence of IBMX. A 7.6 fold induction of insulin secretion was observed in presence of 20 mM glucose (Figure 4C). Importantly, IBMX was able to potentiate insulin secretion. Indeed, a 20.5 fold induction is observed with 20 mM glucose in the presence of IBMX compared to 2.8 mM glucose conditions (Fig. 4C).

### Transplanted RYAS41 cells restore normoglycemia in diabetic mice

**Figure 5:**
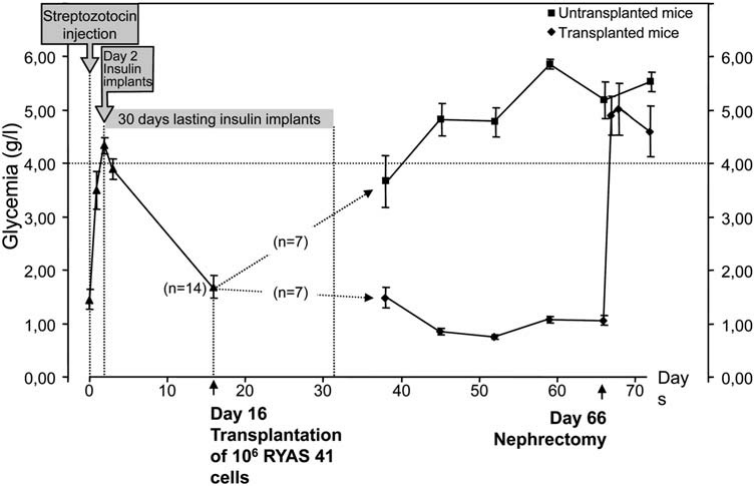
Transplanted RYAS41 cells restore normoglycemia in diabetic mice. Fourteen *scid* mice were injected with streptozotocin. Two days later, 3-weeks lasting insulin capsules were subcutaneously implanted to hyperglycemic mice. Two weeks later, half of the mice were transplanted under the kidney capsule with 10^6^ RYAS41 cells. Grafted cells were removed by nephrectomy at day 66. Values are means+/−S.E.M.

To define whether RYAS41 cells were functional, *scid* mice were injected with streptozotocin for beta cells destruction and two days later, insulin capsules were subcutaneously implanted to hyperglycemic mice to maintain normoglycemia. Sixteen days after STZ injection, half of the mice (n = 7) were transplanted with 10^6^ RYAS41 while the other 7 were used as control. At day 38, insulin release by the implanted capsules lasted and glycemia of non transplanted mice was increased and remained high up to the end of the experiments (72 days), demonstrating the efficacy of beta cell destruction by streptozotocin. On the other hand, glycemia of transplanted mice remained in the normal range (Fig. 5). When unilateral nephrectomies were performed to remove the grafts, mice became hyperglycemic establishing that in streptozotocin-treated mice, glycemia was controlled by the grafted cells. As expected, when transplanted animals were kept for a longer period, severe hypoglycemia systematically occurred 80–90 days after transplantation due to expansion of tumoral RYAS41 cells (data not shown).

## Discussion

Production of human pancreatic beta cells in large amount represents a crucial objective for 2 main reasons: such beta cells would be useful for screening of new drugs that can modulate beta cell function and most importantly, pancreatic beta cells could be used for cell therapy of diabetes. Different approaches have been previously developed to generate pancreatic beta cells in large amount. A first one consisted in using as starting material embryonic stem cells (ES cells) to produce beta cells. The major advantage is that ES cells self-renew indefinitely in culture, and have the capacity to differentiate to multiple cell types, and thus to pancreatic beta cells. Quite a large amount of publications appeared during the past years on beta cell production from mouse ES cells [16] and more recently with human ES cells [17]–[19]. However, mass production of pure beta cells could not be achieved with such process [19].

A second approach to generate large amounts of beta cells was based on the production of pancreatic beta cell lines using adult pancreas as a starting material. First experiments were performed with rodent tissues. The rat cell lines RIN and INS and the hamster cell line HIT were obtained using this approach [1]–[3]. In 2005, a human beta cell line NAKT-15 was produced using human adult islets as starting material [20]. While this cell line seemed functional and stable, to the best of our knowledge, since 2005, no information was published with this line. Thus, while major efforts were developed to generate human beta cell lines from adult islets [20]–[23] only one human beta cell line was developed [20], demonstrating the importance of generating additional human beta cell lines and of defining alternative strategies.

A third approach was to derivate beta cell lines from beta cell tumours derived from transgenic mice expressing SV40T antigen under the control of the insulin promoter. There, the immortalizing gene is expressed in beta cells as early as insulin is transcribed [4]–[9]. Such lines were extremely useful as a model to deeply study mouse beta cells [24]. However, since beta cell lines were obtained by gene transfer in fertilized eggs, its application is restricted to animal models without any possible transfer to human.

Recently, we demonstrated that immature embryonic pancreas transduced with recombinant lentiviral vectors resulted in endocrine cell differentiation and restricted cell type expression of the transgene according to the specificity of the promoter used in the viral construct. Specifically, when eGFP was placed under the control of the insulin promoter, a majority of the developed beta cells expressed eGFP [10]. Thus recombinant lentiviral vectors can efficiently transduce pancreatic progenitor cells and thereby stably modify mature rat pancreatic beta cells. Here, we asked whether such an approach could be used to generate beta cell lines. If that was the case, it would mimic the approach used to generate beta cell lines form transgenic mice [4]–[9]. This will generate a transgenic like organ without gene transfer in fertilized eggs thus enabling such an approach to be used in human.

Here, we demonstrated the efficiency of the approach using rat embryonic pancreases. We first developed an approach to generate insulinoma by transducing rat embryonic pancreatic epithelia with recombinant lentiviral vectors expressing the SV40T antigen under the control of the insulin promoter. Here we show that rat embryonic pancreatic epithelia transduced with SV40T antigen expressing lentiviral vectors systematically develop insulinoma when grafted under the kidney capsule of *scid* mice (38 out of 38). This grafting site has been shown to be permissive for the development of many organs such as ovarian cortex, thyroid, skin and airway [25]–[28]. Importantly, we previously demonstrated that pancreatic beta cells properly develop from human immature embryonic pancreases under such conditions [10], [29], [30]. In this study, we selectively immortalized insulin producing cells using immature embryonic pancreatic bud as starting material. Neither acinar cells nor glucagon-expressing endocrine cells were obtained through this selective process. Furthermore, the derived insulin producing cell line did not express immature endocrine markers such as Ngn3 and Pax4. These cells are mature and express a series of beta cell transcription factors such as Pdx1, Nkx6.1, Pax6 and NeuroD1. They also express molecules crucial for beta cell function such as Pcsk1 and Pcsk2 that participate in the processing of proinsulin to insulin and C-peptide, Abcc8 that codes for the sulfonyurea receptor, Glut2 and glucokinase that are important for glucose stimulated insulin secretion, Snap25 the Synaptosomal-associated protein 25 kDa and Rab3A a small G protein, member of the Rab family and glucokinase[31]. Such molecules were expressed in similar amounts in Ryas41 cells when compared to well characterized 832/13 INS-1 cell line [15]. RYAS41 cells secrete insulin in response to glucose stimulation, but unexpectedly, insulin secretion is highly induced by IBMX at 2.8 mM glucose which is not the case in primary beta cells [32], [33]. Moreover, the percentage of insulin content secreted per hour under stimulated conditions is much higher to what observed with primary islets and beta cell lines [2]. Such differences will be investigated in the future. At this point, RYAS41 cells are not clonal but sub-clones could be established to improve regulated insulin secretion as described for INS-1 cells [24]. Insulin mRNA level and insulin content in RYAS41 and 832/13 INS-1 cell line are similar but remained lower than the ones found in primary beta cells [34]. Finally, upon transplantation, RYAS41 cells regulate the glycemia of NOD/*scid* mice deficient in endogenous beta cells.

Our current objective is to transfer to human the above described validated technology with the goal of generating functional human beta cell lines. Our past and current data support the feasibility of this objective. First, beta cells develop from human fetal pancreas grafted under the kidney capsule of immunoincompetent mice [29]. Next, we recently established conditions for somatic gene transfer in human fetal pancreatic progenitors allowing stable transgene expression into beta cells [35]. Finally, our preliminary data indicate that human insulinoma develop upon SV40T antigen gene transfer into human pancreatic progenitors using the above described approach.

In conclusion, we described in the present work a new approach based on somatic gene transfer applicable to human tissues to generate functional beta cell lines starting from rat embryonic pancreas. This strategy provides an interesting novel route to generate human beta cell lines and also cell lines from other specific cell types using SV40 T antigen under the control of other cell type specific promoters.

## Materials and Methods

### Ethical Statement

All animals were handled in strict accordance with good animal practice as defined by the French animal welfare bodies, and all animal work was approved by the Direction Départementale des Services Vétérinaires de Paris.

### DNA constructs and recombinant lentiviral productions

The backbone of the lentiviral construct, pTRIP, has been previously described [36]. The lentiviral vector, pTRIP ΔU3.RIP405-eGFP expresses eGFP under the control of the Rat insulin II gene promoter (RIP) [10]. New lentiviral vectors pTRIP ΔU3.RIP405-LargeT and pTRIP ΔU3.RIP405 –NEO were constructed in order to express, under the control of the insulin promoter, the SV40T antigen or the neomycin resistance gene respectively. First the eGFP cassette was removed from pTRIP ΔU3.RIP405-eGFP by BamHI and KpnI restriction. The following linker, gatcgccccgggcgggatccggtac with BamHI and KpnI cohesive ends was ligated to the linearized plasmid resulting in the pTRIP ΔU3.RIP405-linker containing downstream of the insulin promoter SmaI, BamHI and KpnI unique cloning sites in the 5′ to 3′ orientation. A BamHI insert containing the entire coding region of the SV40T antigen (kindly provided by B. Thorens) was ligated to a BamHI linearized pTRIP ΔU3.RIP405-linker. The complete coding region of the neomycin resistance gene was amplified from the pcDNA 3 plasmid (Invitrogen, Carlsbad, CA) by PCR using the following primers: BamHI-Neo sense: 5′gaggaggatccCGCATGATTGAACAAGATGG 3′ and KpnI-Neo antisens 5′ cccaaggtaccCGCTCAGAAGAACTCGTCAAG 3′. The resulting PCR product was digested with both BamHI and KpnI and ligated in a BamHI, KpnI linearized pTRIP ΔU3.RIP405-linker. To rule out PCR induced mutations the neomycin resistance coding region was entirely sequenced.

Lentiviral vector stocks were produced by transient transfection of 293T cells with the p8.7 encapsidation plasmid (ΔVprΔVifΔVpuΔNef) [37], pHCMV-G encoding the VSV glycoprotein-G [37] and the pTRIP ΔU3 recombinant vector as previously described [36]. The supernatants were treated with DNAse I (Roche Diagnostic, Meylan, France) prior to ultracentrifugation and the resulting pellet was resuspended in PBS, aliquotted and frozen at −80°C until use. The amount of p24 capsid protein was quantified by the HIV-1 p24 ELISA antigen assay (Beckman Coulter, Villepinte, France). All infections were normalized relative to p24 capsid protein quantification.

### Preparation of pancreatic rudiments, infection and transplantation

Pregnant Wistar rats were obtained from Janvier (CERJ, Le Genest, France). All animal manipulations were performed according to the guidelines of the French Animal Care Committee. The morning post coitum was designated as embryonic day 0.5 (E0.5). Pregnant female rats at E13.5 days of gestation were sacrificed by cervical dislocation. The embryos were harvested on E13.5 and dissected. The dorsal pancreatic bud was dissected and the epithelium was mechanically separated from the surrounding mesenchyme as described [38], [39].

Recombinant lentiviruses were used to transduce embryonic pancreatic epithelia. Briefly, 1 µg of p24 of either pTrip ΔU3.RIP405-eGFP or pTRIP ΔU3.RIP405-SV40T was pre-incubated in a final volume of 45 µl of RPMI 1640 medium supplemented with 10% heat inactivated fetal calf serum containing HEPES (10 mM), L-glutamine (2 mM), non essential amino acid (Invitrogen) and penicillin (100 units/ml)-streptomycin (100 µg/ml) and DEAE-dextran (20 µg/ml). After 15 min at 37°C of pre-incubation the viral solution was added to 45 µl of Hepes Buffered Saline Solution (HBSS, Invitrogen) containing 10 pancreatic epithelia. After 2 hours of infection, tissues were washed twice in culture medium and grown overnight in three-dimensional collagen gels as described previously [38], [39]. The following day, the epithelia were removed from the collagen matrix and used for transplantation into severe combined immunodeficient (*scid*) mice.

Male *scid* mice (Charles River Laboratories, L'arbresle, France) were maintained in isolators. Using a dissecting microscope, 10 infected pancreatic epithelia were implanted under the kidney capsule as previously described [29], [30]. At different time points after transplantation, the mice were sacrificed, the kidney removed, and the graft dissected. Tissues were either fixed and used for immunohistological analysis or for in situ hybridization or used to establish beta cell lines. Some mice were pulsed with BrdU (Sigma-Aldrich, Saint-Quentin Fallavier, France) 2 hour before sacrifice for cell proliferation analysis.

### Graft dissociation and culture and establishment of a beta cell line

Three month after transplantation *scid* mice were sacrificed by cervical dislocation and the grafts were removed, cut into pieces in a sterile cabinet and weighted. and treated with 200 units of type IV collagenase in 500 µl of HBSS during 20 min at 37°C. The digested tissue was centrifuged for 10 min at 2000 rpm and the resulting pellet was resuspended in culture medium containing DMEM, 15% heat inactivated fetal calf serum, 0.5% 2-mercaptoethanol and penicillin (100 units/ml)-streptomycin (100 µg/ml). The suspension was mechanically dissociated in a 1 ml syringe by successive passages through 21, 22, 25, 27 and 30 gauge needles. The dissociated cells were centrifuged 10 min at 2000 rpm and resuspended in a volume of 300 µl of culture medium per 50 mg of initial tissue and seeded on a poly-L-lysine / laminin coated 1.5 cm^2^ culture wells.

After 24 hours of culture, cells were infected at 37°C with 60 ng of p24 capsid protein of pTRIP ΔU3.RIP405 –NEO in 200 µl of culture medium supplemented with 10 µg/ml of DEAE dextran. One hour later, the medium was replaced by 1 ml of fresh medium. G418 (Sigma) was added in the medium at a final concentration of 1 mg/ml 2 weeks after infection. For the first 10 passages the cells seeded in coated wells of either equivalent or double surface in order to achieve a two fold dilution. Passage was performed when cell confluence was observed. From passage 11, a 2/5 dilution was performed every week to amplify the cell line.

### Tissue preparation for histological analysis

Tissue fixation was performed by intracardiac perfusion of 4% paraformaldehyde (PFA) freshly prepared in phosphate buffered saline (PBS). Then, different post fixation procedures were applied. For immuno-detection on paraffin sections perfused tissues were postfixed 6 hours in 3.7% formaldehyde then dehydrated and embedded in paraffin. 4 µm sections were performed and used for immunofluorescent co-detection for both insulin/Pdx1 and insulin insulin/BrdU. For frozen section, the perfused tissues were postfixed for 2 hours in 4% PFA then cryoprotected in 15% sucrose prepared in PBS for 48 hours. The tissues were next embedded in 7% gelatin, 15% sucrose prepared in PBS, frozen at −50°C in isopentan and 10 µm sections were performed. Such tissues were used for immunofluorescent co-detection of insulin/SV40T antigen. For in situ hybridization, cryo-sections were performed on as describe above after a 24 hours postfixation period.

### Immunohistochemical procedures on tissue sections and RYAS41 cell line

Immunofluorescent staining was performed as previously described [38]. For immunofluorescent detection on Ryas 41 cells, 12 mm glass cover slips were coated with poly-L-lysine / laminin in a 1.5 cm^2^ culture well. 1.2 10^5^ RYAS41 cells were seeding and cultured for 5 days. Two hours before fixation a BrdU (10 µM) was added to the culture medium. Next, cells were fixed in 4% PFA.

The following antibodies were used: Rabbit anti-Pdx1 polyclonal antibody (1/1000) [38]; guinea pig anti insulin antibody (1/400, DakoCytomation, Trappes, France); rabbit anti-insulin antibody (1/200, Diasorin, Anthony, France); mouse anti-BrdU (1/2, Amersham, France), and mouse anti-SV40T (1/50, Calbiochem Merck Biosciences, San Diego, CA) and anti-rabbit C-peptide (1/1000, Beta Cell Biology Consortium). The fluorescent secondary antibodies were fluorescein anti-rabbit antibody (1/200; Jackson Immunoresearch Laboratories, Beckman Coulter); fluorescein anti-mouse antibody (1/200, Immunotech, Marseille, France) and Texas-red anti-guinea pig antibodies (1/200; Jackson Immunoresearch Laboratories).

### In situ hybridization coupled with BrdU immunohistochemical detection

The proinsulin probe was prepared as previously described [40]. Plasmids were linearized and used as templates for the synthesis of antisense riboprobes by T3 RNA polymerase (Promega), in the presence of digoxygenin-UTP (Roche diagnostic). Colorimetric revelations were performed with 5-bromo-4-chloro-3-indolyl phosphate (Promega) and nitroblue tetrazolium (Promega) for digoxygenin-UTP.

After in situ hybridization, BrdU incorporation was visualized by immunohistochemical analysis using an anti-BrdU antibody (1∶500; Becton Dickinson) and biotinylated anti-mouse secondary antibodies (1∶200).

### Digital photographs

Photographs were taken either using a fluorescent microscope (Leica; Leitz, Rockleigh, NJ) and digitized using a cooled three-chip charge coupled– device camera (Hamamatsu C5810; Hamamatsu, Middlesex, NJ) or using an Axioskop microscope (Zeiss) and a color vision digital camera (Donpisha).

### RNA isolation, reverse transcription and real-time-PCR

Total RNA was isolated from RYAS41 and 832/13 INS-1 cell lines [15], and lung and pancreas from E17 rat embryos using the Quiagen RNeasy microkit (Quiagen). cDNA was prepared using Superscript (Invitrogen) and Quantitative real-time RT-PCR was performed using assays-on-demand kits and an ABI Prism 7300 sequence detector (both from Applied Biosystems, Foster City, Ca), according to the manufacturer's instructions. For Ngn3, Pax4 and Amylase PCR products obtained after 40 cycles were also loaded on a 2% agarose gel.

### Insulin secretion and insulin contents

RYAS41 cells (3.10^5^ cells) were seeded in coated wells. 3 days later cells were incubated overnight with culture medium containing 2.8 mM Glucose. Cell were next washed twice with HEPES-buffered Krebs-Ringer buffer (KRB) (119 mmol/l NaCl, 4.74 mmol/l KCl, 2.54 mmol/l CaCl_2_, 1.19 mmol/l MgSO_4_, 1.19 mmol/l KH_2_PO_4_, 25 mmol/l NaHCO_3_, 10 mmol/l HEPES at pH 7.4, and 0.1% bovine serum albumin [BSA] [Sigma]). Insulin secretion was measured by using static incubation for a 90-minutes period in KRB containing 2.8 mM or 2.8 mM Glucose supplemented in the presence or absence of 0.5 mM IBMX. Insulin secretion and content were measured by radio-immuno-assay using the Diasorin INSIK-5 kit according to manufacturer instructions

### RYAS41 transplantation in diabetic mice

To determine the ability of the RYAS41 cell line to regulate the glycemia of the diabetic mice, *scid* mice were injected with a solution of Streptozotocin (STZ; 250 mg/kg of body weight; Sigma-Aldrich) freshly prepared in citrate buffer to destroy beta cells. Glucose concentrations were measured on blood collected from the tail vein, using a portable glucose meter (GlucoMen, A. Menarini diagnostics, Firenze, Italy). Two days after STZ injection, mice bearing a blood glucose concentration above 4 g/l were implanted subcutaneously with a 3 week lasting insulin capsule (Sustained Release Insulin Implants; LinShin, Scarborough, Canada) in order to normalize the glycemia before RYAS41 transplantation. Two weeks later, half of the treated mice were transplanted with 10^6^ RYAS41 cells using the following procedure. Briefly, RYAS41 cells were harvested, centrifuged 10 min à 4°C. The cell pellet was resuspended in 12 µl of ice cold matrigel (BD Bioscience) and placed in a silicon cylinder at 37°C to polymerize. Then the cylinder containing the RYAS41 cells was transplanted under the kidney capsule of STZ treated mice. To confirm the contribution of the RYAS41 graft to the normalization of blood glucose values in the host mice, grafts were removed by unilateral nephrectomy at the end of the experiment.
